# Electronic-photonic arithmetic logic unit for high-speed computing

**DOI:** 10.1038/s41467-020-16057-3

**Published:** 2020-05-01

**Authors:** Zhoufeng Ying, Chenghao Feng, Zheng Zhao, Shounak Dhar, Hamed Dalir, Jiaqi Gu, Yue Cheng, Richard Soref, David Z. Pan, Ray T. Chen

**Affiliations:** 1 Microelectronics Research Center, The University of Texas at Austin, Austin, TX 78758 USA; 2 Computer Engineering Research Center, The University of Texas at Austin, Austin, TX 78705 USA; 3 Omega Optics, Inc., 8500 Shoal Creek Boulevard, Building 4, Suite 200, Austin, TX 78757 USA; 4 Department of Engineering, University of Massachusetts Boston, Boston, MA 02125 USA

**Keywords:** Integrated optics, Optoelectronic devices and components, Silicon photonics

## Abstract

The past two decades have witnessed the stagnation of the clock speed of microprocessors followed by the recent faltering of Moore’s law as nanofabrication technology approaches its unavoidable physical limit. Vigorous efforts from various research areas have been made to develop power-efficient and ultrafast computing machines in this post-Moore’s law era. With its unique capacity to integrate complex electro-optic circuits on a single chip, integrated photonics has revolutionized the interconnects and has shown its striking potential in optical computing. Here, we propose an electronic-photonic computing architecture for a wavelength division multiplexing-based electronic-photonic arithmetic logic unit, which disentangles the exponential relationship between power and clock rate, leading to an enhancement in computation speed and power efficiency as compared to the state-of-the-art transistors-based circuits. We experimentally demonstrate its practicality by implementing a 4-bit arithmetic logic unit consisting of 8 high-speed microdisk modulators and operating at 20 GHz. This approach paves the way to future power-saving and high-speed electronic-photonic computing circuits.

## Abstract (web summary)

Integrated photonics allows integration of complex optical circuits on a single chip. Here, the authors propose a wavelength division multiplexing based electronic-photonic arithmetic logic unit for computing at high speeds and with improved power consumption to help with the physical limits of Moore’s law.

## Introduction

The breakdown of Dennard scaling in the early 2000s, when the features began to shrink below around 90 nm, led to the stagnation of the maximum clock frequency up to a few GHz due to the overwhelming heat^1,2^. Since then, the industry has started to focus on multicore processors as an alternative way to improve performance which continues the Moore’s law, a rule of thumb that dominates computing^3,4^. Unfortunately, nowadays Moore’s law has been facing fatal challenges once again as the nanofabrication technique goes to a several-nanometer limit where quantum uncertainties will govern the electron behavior and make transistors unreliable^2,5^. This saturation has forced massive researches in industry and academia on various aspects ranging from nanofabrication technology^6^, material science^7^, computing architecture^8^, as well as new computing mechanisms such as quantum computing^9^.

Integrated photonics is poised to revolutionize traditional electrical interconnects with the trend from long-haul communication links down to inter- and intra-chip connections^10–14^. It is even possible nowadays to achieve a high-performance optical memory-processor link on the same chip with the newly demonstrated “Zero-change” fabrication technique that tailors photonics devices to be integrated directly with transistors using the mature complementary metal–oxide–semiconductor (CMOS) fabrication line^15,16^. As abundant passive and active components mature in integrated photonics^17–20^, electronic-photonic computing (EPC) that uses photons to transport and process information instead of electrons has attracted increasing attention. It includes two directions, analogy computing^21–23^ and digital computing^24^ for various applications. Decades of successful practical experience of very-large-scale-integration (VLSI) have proven the indubitable significance of digital circuits for computing due to the advantages of much better noise tolerance as well as power saving.

It is worth mentioning several facts and limits of transistors in VLSI circuits. Firstly, although the power consumption and latency within the logic gates have been reduced over time, those for transporting signals between gates do not scale down in the same way, and that dominates the performance of the entire system^25,26^. Secondly, the power consumption of a certain transistor-based circuit has a positive correlation with *f*^3^ (*f* is the clock frequency)^27^. In other words, the power grows exponentially as the system clock frequency increases. Fortunately, using light to process information enables us to avoid these obstacles potentially. First, light travels much faster on a chip and normally it only takes a subpicosecond to go through a micron-scale gate which is 1–2 orders of magnitude faster than it takes electrons to go through a transistor with several fanouts^28^. Second, not like the transistors where the voltage required for efficient switching is related to the frequency, optical circuits only consume the power proportional to *f*. Third, Bosons, photons in this case, have the unique property of not abiding by Pauli exclusion principle, creating multiplexing techniques unique to light such as wavelength division multiplexing (WDM) and polarization division multiplexing, which offer the ability to further scale the computing capacity substantially^29^.

Here, we propose a WDM-based electronic-photonic arithmetic logic unit (EPALU) for computing at higher speed and with lower power consumption. We begin with a theoretical proposal of a general EPALU architecture, which makes full use of the advantages of electronics and photonics. We experimentally demonstrate the essential part of our proposed architecture by implementing a 4-bit optical carry propagation network (OCPN) operating at 20 GHz. A thorough analysis of its performances, including power efficiency, computation speed, and insertion loss, shows that such an EPC circuit is capable of running at tens of GHz while only consuming 1–2 orders of magnitude less power than state-of-the-art electronic circuits at a high clock rate due to the non-exponential relationship between power and frequency. Lastly, various scaling methods are discussed to enable optical Moore’s law for EPC.

## Results

### Architecture

**Fig. 1:**
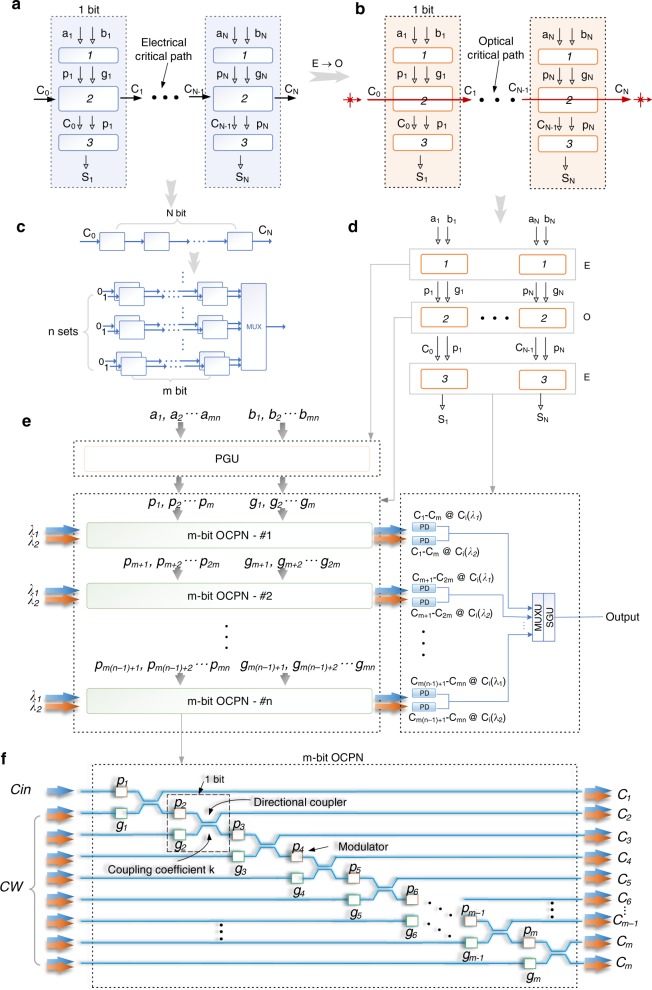
General architecture of the EPALU. **a** Schematic of a conventional transistor-based ripple-carry full adder, with carry signals rippling along the critical path. **b** General schematic of the electronic-photonic full adder. The components along the critical path are replaced by optical ones and light transports the carry signals from one bit to another. **c** Schematic of a ripple carry adder (upper) and a carry select adder (lower). **d** Variant of (**b**), to show the correspondence between (**b**) and (**e**). **e** General architecture of the WDM-based EPALU, consisting of a (p, g) generation unit (PGU), *n* sets of *m*-bit optical carry propagation networks (OCPNs) and an array of fast photodetectors (PDs) along with a network of electronic multiplexer units (MUXU) and an electronic sum generation unit (SGU). **f** Schematic of the *m*-bit OCPN consisting of an optical network with modulators and passive couplers. Different input combinations will lead to different functions.

An ALU that performs arithmetic and bitwise operations such as add, subtract, increment, compare, and logical operations on integer binary numbers is a fundamental building block of various significant computing modules including the central processing units (CPUs), floating-point units (FPUs), and graphics processing units (GPUs). As a key part in an ALU, a full adder adds two addends and one carry signal travels from the first bit to the last along the critical path that finally determines the total latency and thus the operating speed. The expression for a full adder can be summarized by

1 $${\it{C}}_{\it{n}} 	= \left( {{\it{a}}_{\it{n}} \oplus {\it{b}}_{\it{n}}} \right) \cdot {\it{C}}_{{\it{n}} - 1} + {\it{a}}_{\it{n}} \cdot {\it{b}}_ {\it{n}} = {\it{p}}_{\it{n}} \cdot {\it{C}}_{{\it{n}} - 1} + {\it{g}}_{\it{n}}$$

2 $${\it{S}}_{\it{n}} = {\it{C}}_{{\it{n}} - {\mathrm{1}}} \oplus \left( {{\it{a}}_{\it{n}} \oplus {\it{b}}_{\it{n}}} \right) = {\it{C}}_{{\it{n}} - {\mathrm{1}}} \oplus {\it{p}}_{\it{n}}$$

where ${\it{p}}_{\it{n}} = {\it{a}}_{\it{n}} \oplus {\it{b}}_{\it{n}}$ (propagate) and ${\it{g}}_{\it{n}} = {\it{a}}_{\it{n}} \cdot {\it{b}}_{\it{n}}$ (generate). Figure 1a shows the schematic of an *n*-bit ripple carry adder, where the carry signal propagates through the critical path. Significant effort has been made towards optimizing the performance of a full adder by changing the architectures for the past half century and examples include carry-lookahead adders, carry-save adders, and conditional sum adders (CSAs)^30^. The basic idea of CSAs, one type of carry-select adders, is to split the *N* bit full adder (Fig. 1c, upper inset) into *n* sets of *m*-bit full adders (Fig. 1c, lower inset) (*N* = *m* × *n*) and thus all these sets are able to perform the calculation simultaneously to reduce the latency. However, the problem is that the carry signals between adjacent sets are unknown before the computing. Therefore, two sets of hardware are adopted here to generate two sets of outputs with the assumption that the incoming carry signal for each set is zero and one, respectively, since these are all possibilities in a binary system, as shown in Fig. 1c (also see Supplementary Note 1). Once the incoming carry is known, we only need to select the correct set of outputs using multiplexers (MUXs) based on the output carry signal from the previous set without waiting for the carry to further propagate through the entire full adder. The disadvantage is that it needs an extra set of full adder circuits, which consumes more power and more precious space on a chip.

Figure 1b shows a general architecture of the electronic-photonic full adder in which the critical path is replaced by an optical route. Light has 1–2 orders of magnitude less latency per gate than the transistors so that it makes ultrahigh-speed computing possible. Furthermore, light beams with different wavelengths or other properties such as polarization can go through the same structure simultaneously and independently. Therefore, the extra identical set of full adder circuits can be eliminated by using two wavelengths in an optical CSA circuit. Incoming carry signals of one and zero can be encoded in two wavelengths and two sets of results are calculated independently.

As shown in Fig. 1e, an *N*-bit (*N* normally is 8, 16, 32, 64…) EPALU can be decomposed to *n* sets of *m*-bit optical carry propagation network (OCPN) (*N* = *m* × *n*) along with an array of integrated photodetectors and electrical circuits. Its relationship with the general architecture in Fig. 1b regarding each functional block is depicted in Fig. 1d. It is similar to the architecture of electrical CSA shown in Fig. 1c, but here the two sets of circuits with different inputs (0/1) now can be realized by one set of optical routes with two wavelengths and different inputs (0/1) are encoded into these two wavelengths. This decomposition offers two advantages. First, it reduces the total latency, which is based on the performance of optical and electrical gates. Second, it offers a solution to surviving from the loss since there are few efficient and compact integrated-optical amplifiers up till now on some monolithically integration platforms such as the silicon platform. The schematic of the *m*-bit OCPN is depicted in Fig. 1f, which is generated by an automated logic design algorithm (see Supplementary Note 10) and consists of a network of optical modulators and couplers. Two electro-optic modulators along with a 2-by-2 coupler compose one bit. Beams of continuous wave (CW) with different wavelengths are injected into the circuit. Note that they have two different combinations (the input of the first port, Cin port, is different), which represent the carry signals of one and zero. Within each clock cycle, all the electrical signals will be injected into the modulators simultaneously. The coupling efficiency for the couplers is set at 50% for this purpose and could be adjusted to optimize the performance, which will be discussed hereinafter. The generated carry signals emerge at the output ports after filters and will be received by an array of fast photodetectors followed by a network of electronic multiplexer units (MUXU) and a sum generation unit (SGU) (see Supplementary Notes 1 and 2). The overflow of this architecture is discussed in Supplementary Note 3. The entire EPALU including the electronic and photonic parts can be fabricated monolithically on a single chip using modern nanofabrication technology^15^. This EPALU is capable of performing addition, subtract, increment, decrement, compare, bit operation and so forth.

### Experiment

**Fig. 2:**
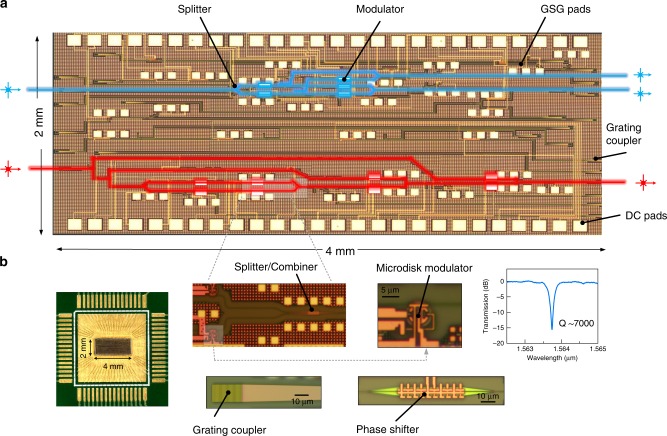
Experimental demonstration. **a** Optical micrograph illustration of the fabricated two-bit (blue) and four-bit (red) OCPN. **b** Micrographs of the wire-bonded chip as well as close-ups of fundamental photonic components such as the phase shifter, the splitter/combiner, the grating coupler, the microdisk modulator, the photodetector, and the crossing. The spectrum of one peak of the microdisk is also shown.

We demonstrated the practicality of the EPALU in integrated silicon photonics by experimentally implementing a 4-bit OCPN. The chip was fabricated by AIM Photonics with over 20 fabrication masks in nanolithography^31^. It is composed of eight high-speed microdisk modulators, with several thermo-optic phase shifters and attenuators located along the paths to maintain the power and phase balance, which are not required in the future fine-tuned system. Note that *p* and *g* signals will not be logic one simultaneously by nature so that no interference between two strong light beams needs to be taken into consideration in an ideal system. While in a practical system where modulators have limited extinction ratio, the phases at each bit can be adjusted to optimize the output. The details and algorithms are discussed in ref. ^32^ (see Supplementary Note 9). The microdisk modulator is chosen as the primary active component due to its compact size and low power consumption^20,33^. Figure 2a shows the micrograph of the fabricated chip with the dimension of 2 mm × 4 mm. The micrograph of the wire-bonded chip and close-ups of the components are also listed in Fig. 2b.

CW light is coupled into the chip through grating couplers and then be split into several portions to be the light inputs for different bits as shown in Fig. 1f. Pseudorandom non-return-to-zero (NRZ) signals are injected through GSG probes after all the static conditions of all the components have been fine-tuned including the accurate wavelength tuning of the microdisk modulators, which needs to align to the operating wavelength. One part of the result signals will be received by integrated photodetectors and the other part will be coupled out of the chip for testing. It should be noted that with the zero-change fabrication technique, it is possible to integrate all the electrical and photonic components onto a single chip^15,16^.

**Fig. 3:**
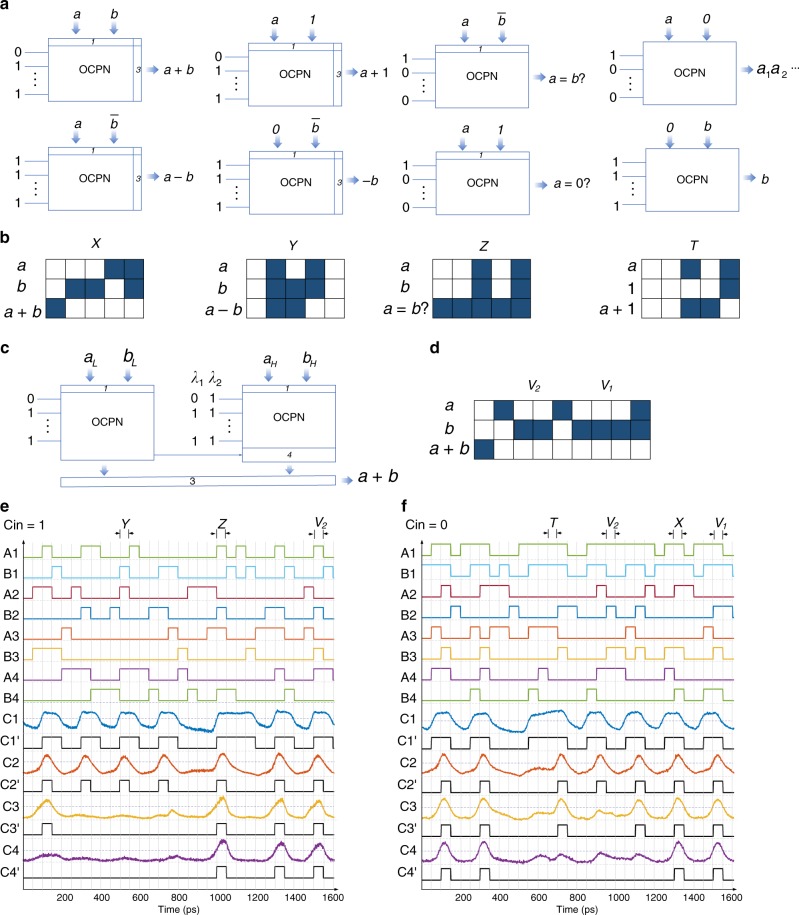
Experimental results. **a** The functions that the proposed EPALU can achieve, including addition, subtraction, increment, decrement, compare and bit operation at different input combinations. Cell 1 represents the PGU. Cell 3 represents the SGU. **b** The testing result of the function realizations by the EPALU. The first two rows are the inputs and the third row is the result. The blue cell means logic 1 and the white cell represents logic 0. The X, Y, Z, T correspond to the time slots in (**e**) and (**f**). **c** Assisted by the WDM, we could obtain results at different input states. As a result, a larger bit size EPALU can be achieved and an 8-bit case is presented. Cell 4 represents the MUXU. **d** One example of the WDM-based 8-bit addition based on the testing result is shown here. V corresponds to the time slot in (**e**) and (**f**). **e** The waveforms of the output carry signals with inputs of pseudorandom NRZ signals injected and Cin equals 1. The operation speed is 20 Gb/s with the operating wavelength of ~1540 nm. **f** The waveforms of the output carry signals with inputs of pseudorandom NRZ signals injected and Cin equals 0. The operation speed is 20 Gb/s with the operating wavelength of ~1565 nm. **f** The waveforms of the output carry signals with inputs of pseudorandom NRZ signals injected. The operation speed is 20 Gb/s with the operating wavelength of ~1565 nm.

Figure 3a shows several functions the EPALU can achieve, including addition, subtract, increment, decrement, compare, and bit operation such as AND with the help of electrical circuits. Different input CW combinations are able to generate different functions, which provides another degree of freedom to manipulate the circuit versatility of function realizations. Several examples of the realized functions in our experiments are shown in Fig. 3b, including addition, subtract, compare, and increment. Take the addition (time slot X) as an example. The operands A = 0011 and B = 1101 will first go through the PGU, marked as cell 1 in Fig. 3a, to generate the P and G signals. Then they will be fed into OCPN to generate carry signals (1111) as shown in Fig. 3f with the assumption that Cin is 0. Finally, the SGU will convert the carry signals into the sum signals (0000) with the Cout of 1. Assisted by the WDM, two sets of outputs at two different carry input states can be obtained at the same time. According to Fig. 1e, larger bit size EPALU can be realized with the help of extra electrical circuits and an 8-bit case is shown in Fig. 3c, d. The waveforms of the carry output C1–C4 at two different operating wavelengths (~1540 and 1565 nm) are depicted in Fig. 3e, f with the operating speed of 20 Gb/s, which are consistent with the truth tables (see Supplementary Note 11).

Note that this architecture of EPALU is suitable for any kind of modulators such as electro-absorption modulator^34^, MZI modulators^17^, microring modulators^35^, plasmonics modulators^36,37^, graphene modulators^38^, and so on. Therefore, the following discussion will be carried out more generally and broadly.

### Computation speed

**Fig. 4:**
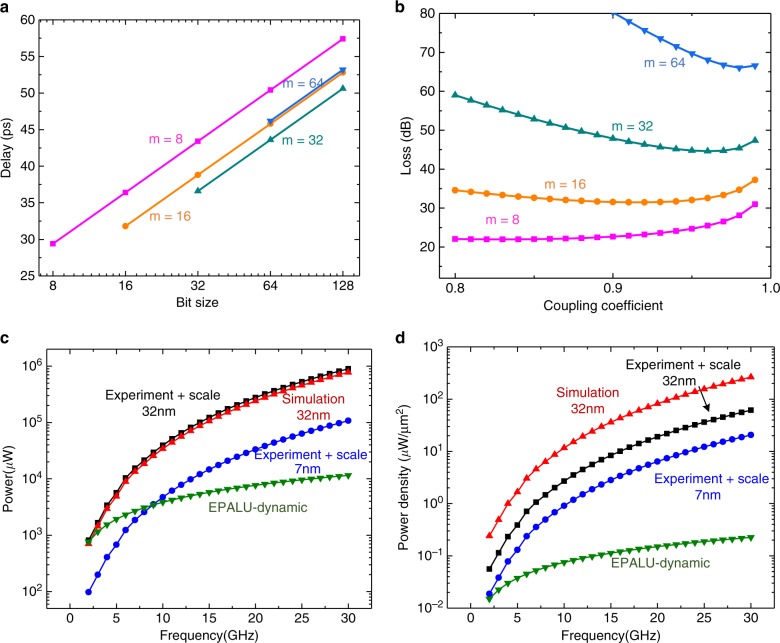
Performance analysis. **a** The entire delay of proposed EPALU with respect to the bit size of the circuit, which also corresponds to the bit size *m* of each optical full adder. **b** The entire loss as a function of the coupling coefficient of the directional coupler. The optimal condition could be obtained for different bit size *m*. **c** Comparison of power consumption between 64-bit EPALU (dynamic power consumption) and conventional transistor-based ALU. The power consumption data on 32 and 7 nm technology node are calculated using the published experimental data (90 nm technology node)^27^ and published accurate scaling equations^38^ as well as commercial simulation tools. Note that the total power consumption of EPALU will be larger if taking laser and thermal tuning into consideration. See details in Supplementary Notes 6 and 7. **d** Comparison of the power density.

The maximum clock rate of the commercial CPUs has stagnated at a few GHz for decades. The proposed EPALU that disentangles the exponential relationship between the frequency and power is promising to escape from the heat death^2^ and breakthrough the computation speed limit. On the other hand, the highly scaled micro-size optical components also enable the light to process information in sub-picoseconds which is 1–2 orders of magnitude faster than electrical gates, leading to a much higher operating speed of the entire circuits. Specifically, as shown in Fig. 1e, the total latency of the EPALU consists of the electro-optic transition time of the modulators ${\it{\uptau }}_{{\it{eo}}}$, the optical propagation latency per gate ${\it{\uptau }}_{\it{o}}$, the optoelectronic transition time of the PDs ${\it{\uptau }}_{{\it{oe}}}$, the electrical latency in the MUXU per stage ${\it{\uptau }}_{\it{e}}$, and the delay for the other electrical parts ${\it{\uptau }}_{\it{g}}$. For an *N* = *m* × *n* bit circuit, the total latency can be expressed as

3 $${\it{\uptau }} = {\it{\uptau }}_{\it{c}} + {\it{m}} \times {\it{\uptau }}_{\it{o}} + {\mathrm{log}}_2{\it{n}} \times \tau _{\it{e}}$$

where ${\it{\uptau }}_{\it{c}} = {\it{\uptau }}_{\it{g}} + {\it{\uptau }}_{{\it{eo}}} + {\it{\uptau }}_{{\it{oe}}}$ is the constant part. Then one can easily optimize the latency based on the values of each parameter in a real platform. Figure 4a shows the entire delay of the EPALU with respect to the bit size with assumptions and details discussed (see Supplementary Note 4). The results indicate that even the 32-bit and 64-bit circuits are capable of operating over 20 GHz under the assumption discussed. It can certainly go faster with the improvement of performances of the active and passive components.

### Energy efficiency

Energy has constrained the design of computing devices in terms of power delivery, battery life, power dissipation, and heat removal^13^. To process data with low power has become a significant challenge. Thanks to the achievement of integrated photonics, various energy-efficient components have been developed and even commercialized in many foundries. For instance, the electro-optic modulator, which is one of the key active components in many applications as well as in our EPALU, now is able to support data rates up to 100 Gbit/s while at the same time consume only femtojoule or even sub-femtojoule power^17,20,36^. Figure 4c shows the curves of power consumption of various circuits as a function of frequency. The power consumption of the EPALU includes that of the optical parts as well as the electrical part (see Supplementary Notes 6 and 7). As a comparison, we also calculated the power consumption of conventional electrical full adder using two methods. First, we use commercial Synopsys Design Compiler to perform the simulation based on the Synopsys Generic 32 nm design library (SAED 32 EDK) which is the most advanced library in the academic area. Second, we use published experimental data based on the 90 nm CMOS technology node from Intel^27^ and estimate the power consumption in 32 and 7 nm technology node using scaling equations^39^. As expected, the power of electrical circuits increases exponentially with respect to frequency. The merits of EPALU start to emerge at a higher frequency and its dynamic power consumption could be 1–2 orders of magnitude smaller than the electrical counterpart when the clock rate exceeds 20 GHz. It should be noted that the total power consumption also includes the static power consumption such as the power of the laser and the thermal tuning. As a result, the total power consumption will be greater than the state-of-the-art 7 nm transistor-based circuit for all frequencies. Fortunately, this part of power can be further reduced or eliminated in the future with the development of photonic components. See detailed discussion of the system power consumption in Supplementary Note 7.

Another important power-related parameter is the power density, the ratio of the power to the area (see Supplementary Note 9), which prevents transistors from going faster owing to overwhelming heat. The comparison of power density is shown in Fig. 4d (also see Supplementary Note 8). It is not surprising to see that the EPALU outperforms the electrical transistors-based circuits here even with one of the most advanced technology nodes since optical components by nature are still larger than transistors.

### Loss

Unlike the transistor-based circuits where signals are automatically normalized to the source voltage or ground, optical signals will also experience optical loss along the paths owing to splitting, insertion loss, and waveguide loss. For example, the carry input from Cin port will encounter 3 dB loss per bit when it propagates to the last Cout port, which is unacceptable for a large-scale circuit. Though an integrated compact amplifier is available in some platforms (e.g., InP platform), it consumes extra power to boost the signals and is not preferred by an energy-efficient computing system. Therefore, the optimization of loss of the EPALU is conducted here by changing the coupling efficiency of the directional coupler. Note that the structure also needs to be revised slightly (see Supplementary Note 5). Figure 4b represents the relationship between the entire loss and the coupling efficiency for OCPNs with 8, 16, 32, and 64-bit size. It indicates that it becomes possible for this large-scale computing circuit to operate freely without the assist of amplifiers. In addition, the choice of *m* and *n* is another dimension we can manipulate to fulfill the loss requirements.

### Scaling and outlook

It is foreseeable to further scale the capacity of the proposed EPALU as well as other photonics-assisted computing circuits at the rate comparable to Moore’s law through at least four directions. First, the performance of the circuits is determined by the components especially the modulators. The progress of these passive and active modules in terms of the size, power consumption, insertion loss, and so forth could directly contribute to the entire system. For example, plasmon-assisted electro-optic modulator^36^ with a dimension of ~4 µm^2^ enables about 90% reduction of the optical propagation delay and 99% reduction of the area compared to the calculation above. Second, the massive multiplexing technologies of light are the most natural and suitable methods for parallel computing. Two wavelengths are adopted in our proposal to dramatically improve the entire performance. More multiplexing technologies can be explored in other applications. Third, special logic gates such as multi-operand logic gates have the potential to further shrink the circuit size and save much power^28,40^. Fourth, though a single optical gate may not beat the transistor in terms of the size and the power consumption, dedicated architectures provide photonics-assisted computing circuits the ability to have a simpler design and win by the number of components. It is because the optimization of electrical circuits for higher performance will bring numerous redundant transistors.

A kind of photonics-assisted computing architecture is proposed with an experimental demonstration at 20 GHz. Computing at a higher speed while with lower power consumption has proved to be possible with the help of light. Advanced fabrication techniques could assist to further integrate electronics and photonics onto a single chip. In addition, equivalent Moore’s law in integrated optical computing is expected to scale the circuit with several directions provided. Emerging optical–electrical–optical devices have the potential to be integrated with the proposed architecture to realize more complex computing functions^41^. Further integration of optical computing units, optical inter/intra-chip optical interconnect^42^, and optical clock distribution^43^ can be explored to realize an entire all-optical computing system.

## Methods

### Electrical simulation

We wrote behavioral Verilog code for adders parameterized by number of bits. Each adder had a single flip-flop stage at the input and a single flip-flop stage at the output. We used Synopsys Design Compiler to perform synthesis and technology mapping on the adders using the Synopsys Generic 32 nm design library (SAED 32 EDK). The tool performed gate sizing and netlist optimizations. We used high and regular threshold voltage cells to optimize for power. The optimization passes of Design Compiler included all three metrics of power, timing, and area. Subsequent optimization steps involved delay reduction through gate sizing and selection of cells with lower threshold voltage.

### Chip implementation and testing

The photonic chip layout was developed and drawn in Cadence Virtuoso and verified using Mentor Graphics Calibre. The chip was a fabrication by AIM Photonics^31^. The 52 pads sitting at two sides are designed for power supply, bias signals, and thermal tuning, which were wire-bonded to a printed circuit board. Amplified spontaneous emission lasers from Thorlabs and optical spectrum analyzers from AssetRelay were used for optical characterization and wavelength alignment. Standard single mode fibers (SMF28) were used to couple light into and out of the photonic chip through grating couplers. The minimal coupling loss (~5 dB per facet) was achieved at 8° off-normal from the surface of the chip. The *Q* factors of the microdisk modulators were estimated to be ~7000 by applying a Lorentzian fitting to the transmission spectrum. Peaks of the microdisk modulator are lying at ~1540 and ~1565 nm so that the operating wavelengths are also set around them, respectively. Thermal tuning is performed to all the microdisk modulators to make sure all the wavelengths are well aligned to the laser wavelength. A tunable laser from ID Photonics was used after the static characterization and alignment. The output light was coupled out to an Agilent DCA 86100C with a 30 GHz optical module (Agilent 86109A). Erbium-doped fiber amplifiers (JDSU Oprel EDFAs) were also used to boost the signals followed by an optical tunable filter (Santec, OFT-920). The Agilent E8257D PSG signal generator was used to generate the clock and feed it to the Agilent E8404A VXI mainframe. Non-return-to-zero (NRZ) signals were generated by the two independent N4872A slots with internal delay controls. We used high-bandwidth radio-frequency probes (GGB, 100 µm pitch) to inject the high-speed signals to the chip through GSG pads with the size of 60 × 60 µm^2^ and the pitch of 100 µm.

## Supplementary information

Supplementary Information

## Data Availability

The data that support the findings of this study are available from the corresponding author upon reasonable request.
