# Supplementary material for: Electronic-photonic arithmetic logic unit for high-speed computing
Source: Nat Commun. 2020 May 1;11:2154. doi: 10.1038/s41467-020-16057-3 (PMC7195421; doi:10.1038/s41467-020-16057-3)
Supplement: Supplementary file 1 — Supplementary Information [file 41467_2020_16057_MOESM1_ESM.pdf]

Supplementary Information for

Electronic-photonic arithmetic logic unit for high-speed computing

Zhoufeng Ying [1], Chenghao Feng [1], Zheng Zhao [2], Shounak Dhar [2], Hamed Dalir [3], Jiaqi Gu [2], Yue Cheng[1], Richard Soref [4], David Z. Pan [2], Ray T. Chen [1, 3*]

[1]*Microelectronics Research Center, The University of Texas at Austin, Austin, Texas 78758, USA*

[2]*Computer Engineering Research Center, The University of Texas at Austin, Austin, Texas 78705, USA*

[3]*Omega Optics, Inc., 8500 Shoal Creek Blvd., Bldg. 4, Suite 200, Austin, TX 78757, USA.*

[4]*Department of Engineering, University of Massachusetts Boston, Boston, Massachusetts 02125, USA*

**Corresponding author: chenrt@austin.utexas.edu*

Supplementary Note 1: Circuit diagram of the MUXU

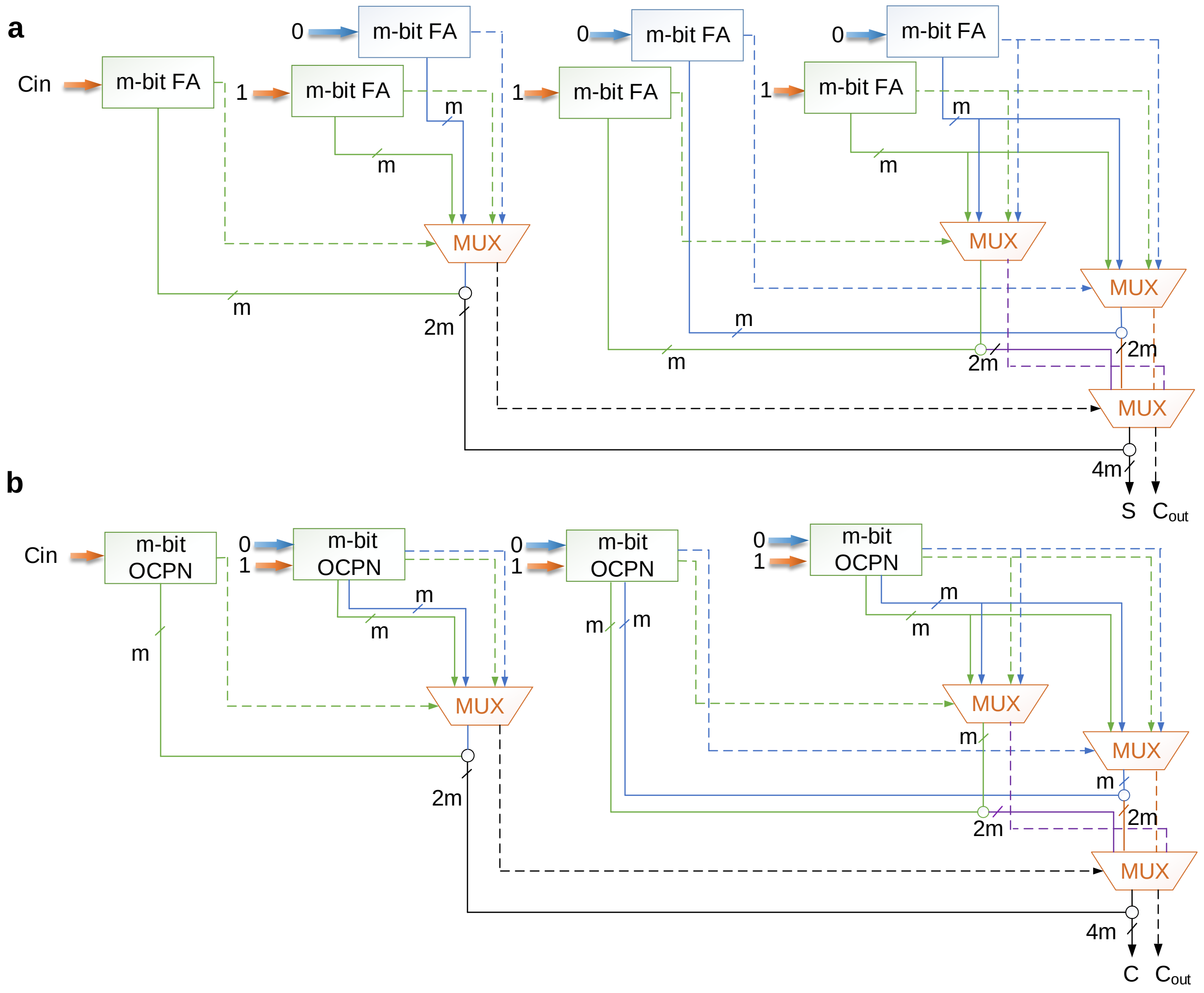

Supplementary Figure 1. Circuit diagram of the MUXU. a. The circuit diagram of the conventional, all-electronic m×4 conditional sum adder, which utilizes two sets of m-bit full adders to calculate the two cases of different input carry signals "1" and "0". b. The circuit diagram of the "photon-assisted" MUXU for the EPALU. It needs only one set of the hardware to do the calculation with the assistance of two-λ WDM. The optimized electrical MUXU has the minimal delay of $\log_2(n)$ and here n=4.

After receiving the optical outputs of the $m \times n$ WDM-based OCPNs and converting them into electrical signals, we need to select the correct set of results based on the carry signal from the

previous *m*-bit OCPN. The worst case is to make the selection from the first bit to the last one-by-one, which has the time complexity of $O(n)$. It can be further improved to $O(\log n)$ when a tree selection unit is adopted. Supplementary Figure 1a shows the case for the 4×m conventional electronic conditional-sum adder with two stages of MUXs. In this architecture, two hardware sets of m-bit full adders are functioning to calculate the two cases of different input carry signals. As a comparison, only one set is required in the EPALU, as shown in Supplementary Figure 1b, with the assistance of WDM. The tree-type MUXU remains the same with the conventional conditional-sum adder and the minimal latency is $\log_2(n)$, which is used in the calculation hereinafter.

Supplementary Note 2: Circuit diagram of the PGU and SGU

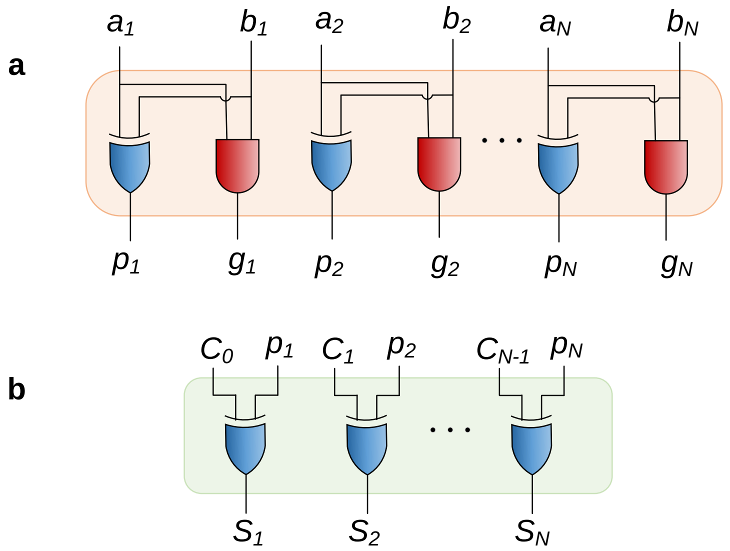

Supplementary Figure 2. Circuit diagram of the PGU and SGU. a. Schematic of the (p, g) generation unit, consisting of XOR gates and OR gates. B. Schematic of the sum generation unit, consisting of XOR gates.

The electronic circuit diagrams of the PGU and SGU are shown in Supplementary Figure 2a and 2b, respectively.

Supplementary Note 3: Overflow

Arithmetic operations such as addition and subtraction have a potential to run into a condition known as overflow. Therefore, an overflow detector is required after the calculation, whose input ports include input operands, output carry signals, and the operation code. For example, the overflow in a signed addition is the XOR of the carry signals of the last two bits before it interacts with the operation code [1]. The operation code means the instruction which determines the function (addition, subtraction, and so on) to perform in the circuit. Fortunately, a traditional electrical overflow detector will fit in this EPALU well since all these required signals are in the electrical domain.

Supplementary Note 4: Latency analysis

The total latency of the EPALU can be expressed as $\tau = \tau_c + m \times \tau_o + log_2 n \times \tau_e$, where $\tau_c = \tau_g + \tau_{eo} + \tau_{oe}$ is the constant part, τ_{eo} is the electro-optic transition time of the modulators, τ_o is the optical propagation latency per gate, τ_{oe} is the opto-electronic transition time of the PDs, τ_e is the electrical latency in the MUXU per stage, and τ_g is the delay for the other electrical parts. With the assumption that τ_g and τ_e for electrical gates are both 7 ps [2], τ_{eo} and τ_{oe} for OE/EO conversion are both 10 ps[3,4], τ_o is 0.3 ps[5], we can obtain the curves in Figure 4a. All these values will vary with platform and fabrication node that are adopted. They could be further reduced as technology advances.

Supplementary Note 5: Loss analysis

To minimize the loss of the circuit, the coupling coefficient could be fine-tuned with the slightly revised structure shown in Supplementary Figure 3a with the one-bit diagram shown in Supplementary Figure 3b. It is obvious that the critical path is the lossiest one which goes through all the couplers. A relatively higher coupling efficient will contribute to the reduction of insertion loss. To be specific, more light should be coupled from port 1 to port 4 or 6. Port 5 is a tap path and only a small portion is required so that we treat the coupling coefficient h to be negligible. Considering the splitting loss, propagation loss, and device insertion loss, we can get the total ratio of the entire input power (all input ports) to the output at the last port, which is

$$\rho = \frac{r^{m-1}-1}{r-1} \times s + r^m,$$

where $r = t^{-1}k^{-1}$, $s = t^{-1}(1-k)^{-1}$, t is the transmission coefficient per bit with all the loss considered. Then the total loss can be calculated as shown in Figure 4b when $t = 0.88$. This value is chosen specifically for the power consumption calculation in Supplementary Note 7.

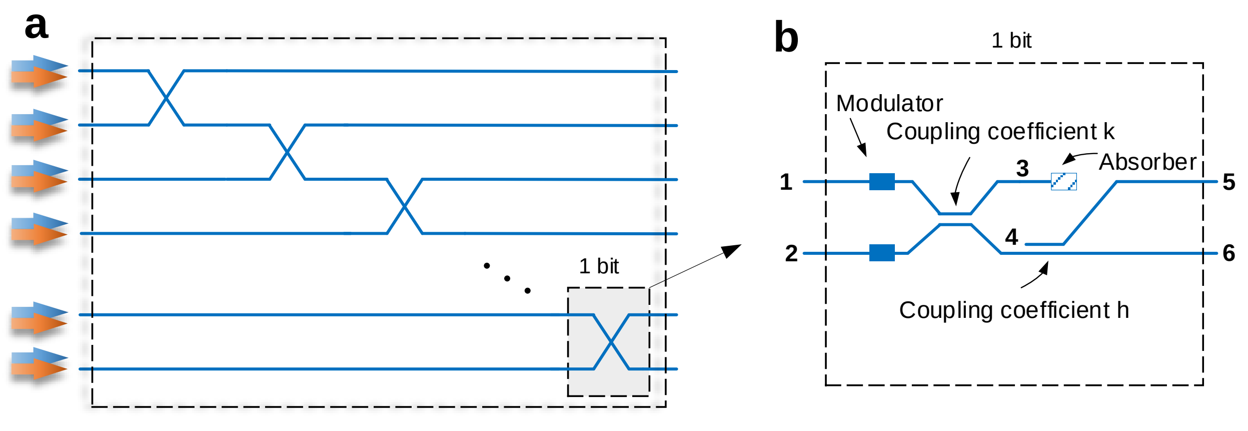

Supplementary Figure 3. Network for loss optimization. a. The network of the OCPN. b. The diagram of the revised structure of one bit in the OCPN.

Supplementary Note 6: Scaling of dynamic power consumption

Our estimation is based on the experimental demonstration of a 64-bit ALU using the 90 nm technology node [6]. We first estimate the power consumption of the ALU part out of the entire chip by calculating the area ratio based on the reasonable assumption that the power consumption is proportional to the area. Along with the scaling equation[2], we can write the scaling equation of power consumption as $\mathrm{P} = \mathrm{P}_0\beta S$, where the P_0 is the power consumption of the entire chip, β is area ratio and S is the scaling ratio. The activity coefficient is 10% in the experiment and we also use this value in our calculation hereinafter. Further, the power increases exponentially with the frequency. From the experimental data in reference [6], we could obtain the relationship between the frequency and the required voltage as well as predict the supply voltage needed at a higher frequency by linearly fitting. Therefore, we could easily predict the power consumption based on the equation of $P \propto CV^2 f$.

The power consumption for each modulator in the proposed EPALU is

$$P = (E_p + E_G + E_M)\alpha f = 3CV^2 f\alpha.$$

The definitions of the related coefficients in the equation are list in the Supplementary Table 1 with the assumption that the inputs *a* and *b* are random signals.

Assuming the capacitance is 10 fF, and swing voltage is 1 V, we have the dynamic power consumption $P = 384CV^2 f\alpha$ for a 64-bit ALU.

Supplementary Table 1. Definition of the variables in calculating the power consumption

Variable	Definition	Value
E_P	Energy for generating P	$E_P = \frac{14}{3} CV^2 \alpha_p$
α_P	Activity coefficient for P	$\frac{1}{2}$
E_G	Energy for generating G	$E_G = \frac{14}{3} CV^2 \alpha_G$
α_G	Activity coefficient for G	$\frac{3}{8}$
E_M	Energy for modulators	$E_M = \frac{1}{4} CV^2$
α	Activity coefficient for ALU	10%

Supplementary Note 7: Total power consumption calculation

In this section, we will carry out a systematic calculation of the power consumption, which includes not only the dynamic part but also the laser part and the thermal tuning part for a microresonator-based EPALU. The estimation is based on the characteristics of the state-of-the-art photonic components.

First, the laser power will be largely determined by the loss as we calculated in Supplementary Note 5. Upon the optimistic assumption that the waveguide propagation loss is 0.002 dB/bit (1 dB/cm), the insertion loss of directional coupler is 0.05 dB, the insertion loss of grating/edge coupler is 1 dB, and the insertion loss of modulator is 0.5 dB [7], we can get total loss for a 64-bit EPALU (e.g. m×n = 8×8) will equal to 21.97 dB (from Fig. 4b) + 9 dB (n is 8) + 3 dB (two wavelengths) + 1 dB (input coupler) = 34.97 dB. When the minimal detectable power is 1 uW [8,9] and the wall-plug efficiency of laser is up to 20% [10], then the total laser power consumption will be 15.7 mW.

Second, microresonator-based modulators will require additional thermal tuning. This part will consume around 50 fJ/bit for each microresonator in a state-of-the-art monolithic electronic-photonic platform [11]. Then in total it requires 50 fJ/bit × 20 Gbit/s ×128 = 128 mW.

As we can see from Supplementary Figure 4, the total power consumption will be dominated by the thermal tuning. This portion of power consumption can be further reduced or even eliminated in the future using broadband modulators (e.g. electro-absorption modulators [12] or low-Q microresonator modulator [13]) or using energy-efficient tuning materials (e.g. phase change material [14]) or more power-efficient methods (e.g. post-fabrication trimming[15]).

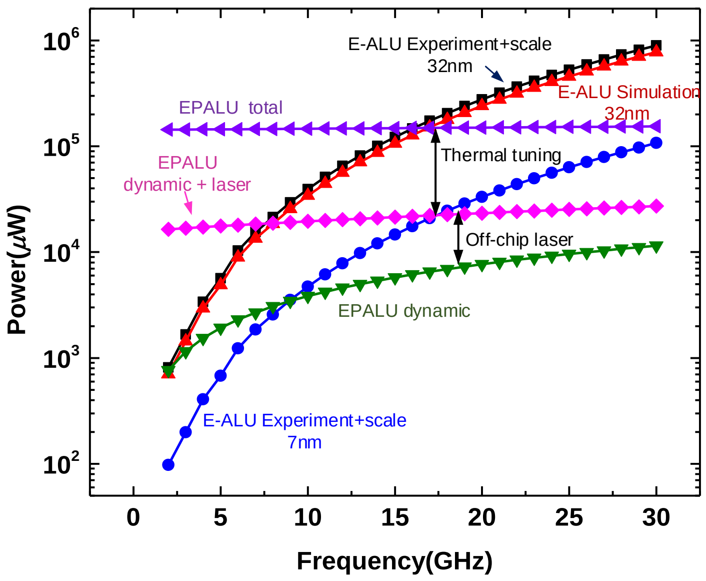

Supplementary Figure 4. Power consumption comparison between EPALU and conventional electronic ALU (E-ALU).

In this 4-bit experiment, the power consumption per bit of the modulator is estimated to be 10.88 fJ/bit since the swing voltage is 0.8 V and the capacitance of the microdisk modulator is estimated to be 17 fF. The laser power is around 4 mW after eliminating the grating coupling loss. The requirement of the relatively higher laser power in the testing is because the coupling coefficients of the directional couplers have not been optimized yet in this first-generation chip/design. The optical signal (>100 μW) is then coupled out for off-chip detection. The resistance of the heaters is 6.03 kΩ, and around 2V is required for wavelength alignment of each microdisk modulator.

Supplementary Note 8: Power density calculation

The crucial part of the estimation of power density is the area calculation. VLSI simulation could directly output the area value, which is 2936.6 μm^2 for a 64-bit full adder. The 'experimental + scale' data are based on the area value from the reference [6] and the area scaling equation [2]. Assuming that each bit of the EPALU is 20×40 μm^2, we can estimate the area of the entire circuit to be 51200 μm^2. Since the laser is off-chip, the wall-plug efficiency of the laser should not be taken into consideration. Therefore, we cross out 80% of the laser power consumption in this calculation. The result is shown in Supplementary Figure 5. Again, thermal tuning will dominate the total power consumption here, which can be further reduced or even eliminated in the future using broadband modulators (e.g. electro-absorption modulators [12] or ultralow-Q microresonator modulator [13]) or using more efficient tuning materials (e.g. phase change material [14]) or more power-efficient methods (e.g. post-fabrication trimming [15]).

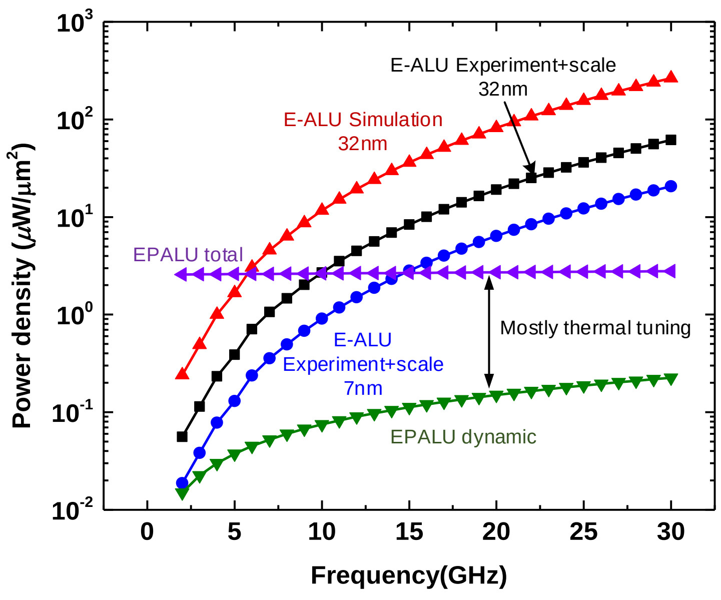

Supplementary Figure 5. Power density comparison between EPALU and conventional electronic ALU (E-ALU).

Supplementary Note 9: Additional discussions

Nowadays the dimensions of photonic components are still in the range of micrometer. As a comparison, transistors have been evolving from micrometer size to nanometer size during the past decades as Moore's law continues, which makes transistors have much higher area efficiency. For example, as indicated in Supplementary Note 8, the proposed EPALU will be 17.4 times larger based on the optimistic estimation. However, as mentioned in the Scaling and Outlook part in the paper, there are many ways to further scale down the size of the photonic circuit in the near future, which has the potential to make ultracompact photonic circuit possible. On the other hand, there are also some applications that are less sensitive to the chip area such as data centers.

The performance of the EPALU significantly depends on the characteristics of the modulators. A modulator with lower power consumption, higher bandwidth, larger extinction ratio and lower insertion loss is ideal for optical computing. Limited performance such extinction ratio of a single modulator will become an obstacle of the whole circuits. The theoretical analysis of impact of the extinction ratio has been discussed in the reference [16].

Supplementary Note 10: Automated logic design

Moore's law has been going on for several decades and nowadays a single semiconductor chip can have billions of transistors. Electronic design automation (EDA) has become an essential part of integrated circuit design. Similarly, as integrated photonic components mature, electronic-photonic design automation (EPDA) has attracted more and more attention in both academia and industry. Many algorithms have been proposed to design these electronic-photonic computing (EPC) circuits. In this paper, the design can also be generated by the And-Invertor-Graph (AIG) algorithm, as shown in Supplementary Figure 6a and 6b. Given Equation 1, this algorithm will

first convert it into an AIG, which is generated by a state-of-the-art open-source logic synthesis tool ABC[17]. After optimizing the number of the AIG node by the embedded AIG-rewriting/balancing techniques[18], this algorithm will start to map each AIG node to the equivalent optical gates in the library. There are two types of gates used in this mapping and the mapping table is listed in Supplementary Figure 6c and 6d. More details of this algorithm can be found in the reference [19]. Note that other algorithms can also be used to generate the logic circuits automatically including the binary decision diagram (BDD) [20].

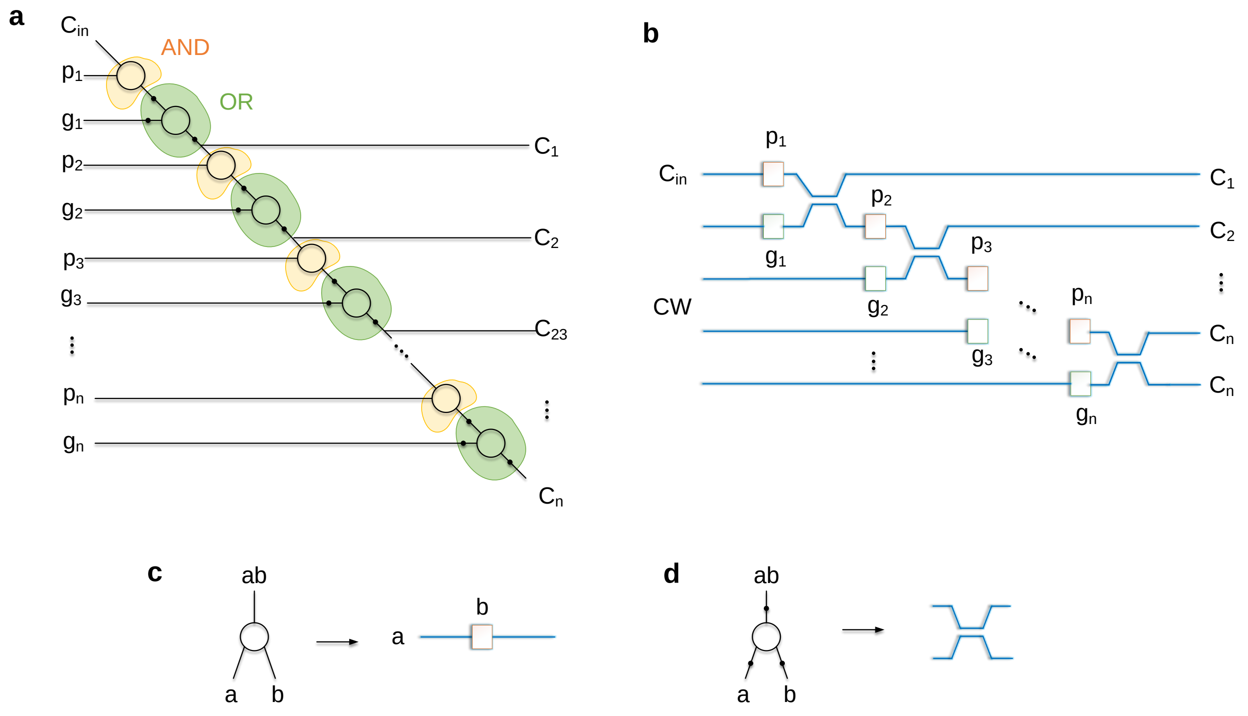

Supplementary Figure 6. Automated design of OCPN. a. And-Invertor-Graph (AIG) representing the equations of carry propagation in a full adder. b. The optical implementation. c, d. The mapping library.

Supplementary Note 11: Truth tables

Supplementary Table 2 and Supplementary Table 3 show the truth tables for the testing results in Figure 3e and 3f, respectively.

Supplementary Table 2. Truth table for Cin=1

Input (Cin = 1)								Output			
A1	B1	A2	B2	A3	B3	A4	B4	C1	C2	C3	C4
0	1	0	0	0	0	0	0	0	0	0	0
0	0	1	0	0	1	0	0	0	0	0	0
1	0	1	0	0	1	0	0	1	1	1	0
0	1	0	0	0	1	0	0	1	0	0	0
0	0	0	0	1	0	1	0	0	0	0	0
1	0	0	1	0	0	1	0	1	1	0	0
1	0	0	0	0	0	0	1	1	0	0	0
0	0	0	0	0	0	0	1	0	0	0	0
0	0	0	1	0	0	0	1	0	0	0	0
0	1	1	0	0	0	1	0	1	1	0	0
1	0	0	0	0	0	1	0	1	0	0	0
0	0	0	0	0	0	1	0	0	0	0	0
0	1	0	1	0	0	0	0	1	1	0	0
0	1	0	0	1	0	0	0	1	0	0	0
0	0	0	0	0	1	1	0	0	0	0	0
0	0	1	0	0	0	0	1	0	0	0	0
0	0	1	0	0	0	0	0	0	0	0	0
0	0	1	0	1	0	0	0	0	0	0	0
1	0	0	1	1	0	0	1	1	1	1	1
0	1	0	0	0	0	0	1	1	0	0	0
1	0	0	0	0	0	0	0	1	0	0	0
0	0	0	0	1	0	0	0	0	0	0	0
0	0	0	1	1	0	0	0	0	0	0	0
1	0	0	1	1	0	1	0	1	1	1	1
1	0	0	1	0	1	1	0	1	1	1	1
0	1	0	0	0	0	1	0	1	0	0	0

Supplementary Table 3. Truth table for Cin=0

Input (Cin = 0)								Output			
A1	B1	A2	B2	A3	B3	A4	B4	C1	C2	C3	C4
0	1	0	0	0	0	0	0	0	0	0	0
1	1	0	0	1	0	1	0	1	0	0	0
1	1	1	0	0	1	1	0	1	1	1	1
0	0	0	1	0	0	0	0	0	0	0	0
1	0	0	0	0	0	0	0	0	0	0	0
1	1	0	0	1	0	0	1	1	0	0	0
0	0	1	0	1	0	0	0	0	0	0	0
0	1	1	0	1	0	0	0	0	0	0	0
1	1	0	0	1	0	0	0	1	0	0	0
1	1	0	1	0	1	0	0	1	1	1	0
0	0	0	0	0	0	0	0	0	0	0	0
1	1	0	0	0	0	0	1	1	0	0	0
1	1	1	0	0	0	1	0	1	1	0	0
1	0	0	1	0	1	1	0	0	0	0	0
1	0	0	0	0	1	1	0	0	0	0	0
1	0	1	0	0	0	0	0	0	0	0	0
1	1	0	0	0	1	1	0	1	0	0	0
1	1	1	0	0	1	0	1	1	1	1	1
0	1	1	0	0	0	0	0	0	0	0	0
1	1	0	1	0	1	0	1	1	1	1	1
0	1	0	1	0	0	0	0	0	0	0	0

Supplementary References

1. Overflow. Available at: http://www.c-jump.com/CIS77/CPU/Overflow/lecture.html.
2. Stillmaker, A. & Baas, B. Scaling equations for the accurate prediction of CMOS device performance from 180 nm to 7 nm. *Integr. VLSI J.* **58**, 74–81 (2017).
3. Reed, G. T. *et al.* Recent breakthroughs in carrier depletion based silicon optical modulators. *Nanophotonics* **3**, 229–245 (2014).
4. Vivien, L. *et al.* Zero-bias 40Gbit/s germanium waveguide photodetector on silicon. *Opt. Express* **20**, 1096 (2012).
5. Ying, Z. *et al.* Electro-Optic Ripple-Carry Adder in Integrated Silicon Photonics for Optical Computing. *IEEE J. Sel. Top. Quantum Electron.* (2018).
6. Mathew, S. K. *et al.* A 4-GHz 300-mW 64-bit integer execution ALU with dual supply voltages in 90-nm CMOS. *IEEE J. Solid-State Circuits* **40**, 44–50 (2005).
7. Timurdogan, E. *et al.* An ultralow power athermal silicon modulator. *Nat. Commun.* **5**, 4008 (2014).
8. Nozaki, K. *et al.* Photonic-crystal nano-photodetector with ultrasmall capacitance for on-chip light-to-voltage conversion without an amplifier. *Optica* **3**, 483 (2016).
9. Nozaki, K., Shinji, M., Akihiko, S. & Masaya, N. Amplifier-Free Bias-Free Receiver Based on Low-Capacitance Nanophotodetector. *IEEE J. Sel. Top. Quantum Electron.* **24**, 1–11 (2018).
10. Wang, H. *et al.* High-Power Wide-Bandwidth 1.55-μm Directly Modulated DFB Lasers for Free Space Optical Communications. *2019 Conf. Lasers Electro-Optics, CLEO 2019 - Proc.* 3–4 (2019). doi:10.23919/CLEO.2019.8750482
11. Wade, M. *et al.* A Bandwidth-Dense , Low Power Electronic-Photonic Platform and Architecture for Multi-Tbps Optical I / O. in *European Conference on Optical Communication (ECOC)* 1–3 (2018).
12. Srinivasan, A. *et al.* 50Gb/s C-band GeSi Waveguide Electro-Absorption Modulator. *Opt. Fiber Commun. Conf.* **1**, Tu3D.7 (2016).
13. Haffner, C. *et al.* Low-loss plasmon-assisted electro-optic modulator. *Nature* **556**, 483–486 (2018).
14. Jiang, W. Nonvolatile and ultra-low-loss reconfigurable mode (De)multiplexer/switch using triple-waveguide coupler with Ge2Sb2Se4Te1 phase change material. *Sci. Rep.* **8**, 1–12 (2018).
15. Atabaki, A. H., Eftekhar, A. A., Askari, M. & Adibi, A. Accurate post-fabrication trimming of ultra-compact resonators on silicon. *Opt. Express* **21**, 14139–14145 (2013).
16. Feng, C., Pan, D. Z. & Chen, R. T. Power and accuracy co-optimization of an optical full

adder via optimization algorithms. *2019 IEEE Photonics Conf.* 1–2 (2019).

17. Mishchenko, A. ABC: a system for sequential synthesis and cerification. Available at: https://people.eecs.berkeley.edu/~alanmi/abc/abc.htm.

18. Mishchenko, A., Chatterjee, S. & Brayton, R. DAG-aware AIG rewriting: a fresh look at combinational logic synthesis. in *2006 43rd ACM/IEEE Design Automation Conference* 532–535 (2006). doi:10.1109/DAC.2006.229287

19. Ying, Z. *et al.* Automated logic synthesis for electro-optic logic-based integrated optical computing. *Opt. Express* **26**, 28002–28012 (2018).

20. Zhao, Z. *et al.* Logic synthesis for energy-efficient photonic integrated circuits. in *Proceedings of the 23rd Asia and South Pacific Design Automation Conference* 355–360 (2018).
